# Public employees in South-Western Norway using an e-bike or a regular bike for commuting – A cross-sectional comparison on sociodemographic factors, commuting frequency and commuting distance

**DOI:** 10.1016/j.pmedr.2019.100881

**Published:** 2019-04-29

**Authors:** Anette B. Jahre, Elling Bere, Solveig Nordengen, Ane Solbraa, Lars Bo Andersen, Amund Riiser, Helga Birgit Bjørnarå

**Affiliations:** a Department of Public Health, Sport and Nutrition, Faculty of Health and Sport Sciences, University of Agder, Servicebox 422, NO-4604 Kristiansand, Norway; b Department of Health and Inequalities, Centre for Evaluation of Public Health Measures, Norwegian Institute of Public Health, Servicebox 222 Skøyen, 0213 Oslo, Norway; c Western Norway University of Applied Sciences, Faculty of Education, Arts and Sport, Post Box 133, 6851 Sogndal, Norway; d Department of Sport Medicine, Norwegian School of Sport Sciences, Servicebox 4014 Ullevål stadion, 0806 Oslo, Norway

**Keywords:** E-bike, electric bicycle, PA, physical activity, MVPA, moderate-to-vigorous physical activity, Agder, Aust-Agder and Vest-Agder counties, E-bike, Bicycle, Active travel, Active commuting, Public employees, Southern Norway, Western Norway

## Abstract

Large-scale analyses on the travel behavior of e-bikes are scarce, and current knowledge regarding who the e-bike owners are is inconsistent. Also, commuters represent a relevant user group with an unexploited potential. Therefore, the purpose of the present study was to examine (i) associations between type of bike (e-bike vs. regular bike) with place of residence (county), sociodemographic variables (age, sex, educational level, income and ethnicity) and habitual physical activity level, and (ii) if public employees possessing an e-bike cycle more often and longer distances to work. A cross-sectional survey was conducted in 2017 among 1977 (5.2% of eligible subjects) public employees in Southern and Western Norway. Binary and multinomial logistic regression analyses were conducted. Respondents possessing an e-bike were less likely to perform high levels of leisure time physical activity (OR 0.56 (CI 0.39-0.82)), compared to those possessing a regular bike only. For those residing in Agder, the likelihood of possessing an e-bike (vs. regular bike) was almost 4 times higher (OR 3.98 (CI 2.53-6.26)), compared with participants residing in Sogn og Fjordane. Compared with those possessing a regular bike only, e-bike users cycled more frequently to work, both occasionally (OR 3.71 (CI 2.44-5.65)) and most of the time (OR 4.28 (CI 2.79-6.55)), and they had higher odds of cycling medium distances to the workplace (OR 1.74 (CI 1.04-2.90)). In conclusion, e-bike access could result in increased commuter cycling, both in terms of cycling frequency and cycling distance, which in turn could contribute to enhanced physical activity levels.

## 1 Introduction

Electric bicycles (e-bikes) have become increasingly popular, and in Europe sales numbers increased from 588.000 in 2010, to 1.667.000 in 2016 (CONEBI, 2017). In Norway, e-bikes accounted for about 10% of the total bike market in 2016, with close to 40.000 e-bikes sold (Tronstad, 2017). Compared to a regular bike, transport by e-bike is faster and less intensive, i.e. less minutes in physical activity (PA) per trip*.* However, the intensity still reaches moderate-to-vigorous physical activity (MVPA) in both healthy adults and patient groups (Bourne et al., 2018), i.e. sufficiently intensive to promote health (Garber et al., 2011). E-bikes could level out common barriers to cycling like hilly terrain, time-use and other practical obstacles, e.g. change of clothes and showering (Dill and Rose, 2012; Fyhri and Sundfør, 2014; Gojanovic et al., 2011; Langford et al., 2017). Also, in competition with motorized modes such as public transport and rush-time driving, the e-bike offers competitive speed, entailing a potential to replace a substantial amount of car and public transport trips (Fyhri and Fearnley, 2015).

Worries have been raised that e-biking will mostly replace other non-motorized travel, i.e. cycling and walking (Fyhri and Fearnley, 2015). Opposing this, it has been observed that in car-dominated countries (e.g. Australia, US and Canada), mainly car trips are replaced (Dill and Rose, 2012; Johnson and Rose, 2013; MacArthur et al., 2013; Popovich et al., 2014). Nonetheless, car use has been found to be significantly higher among e-bike users than non-users, suggesting that e-bikes are used as an additional transport option (Simsekoglu and Klöckner, 2018). Also, higher car use among e-bike users may indicate higher mobility needs in general, as the percentage of households with five or more people is found to be nearly twice as high in e-bike users (Simsekoglu and Klöckner, 2018). In European countries with a bicycle orientation (e.g. The Netherlands and Denmark), the e-bike seems to substitute the conventional bike in addition to the car (Haustein and Møller, 2016; Jones et al., 2016; Lee et al., 2012). However, Kroesen (2017) analyzed data from national mobility surveys in the Netherlands and revealed longer travel distances among e-bike users compared to those riding regular bikes. Accordingly, Fyhri and Fearnley (2015) provided a sample in Norway with e-bikes for two or four weeks, resulting in considerably increased cycling expressed as number of trips, distance cycled, and as cycling shares. Also for the elderly, recent Flemish data support increased probability of cycling, and of higher cycling volumes, in e-bike users (Van Cauwenberg et al., 2018a, Van Cauwenberg et al., 2018b).

As commuting is a cyclical and repetitive activity, commuting by active modes such as e-biking allows for incorporation of PA into daily routines. Hence, commuting by e-bike may contribute to enhanced PA levels, and thereby increased adherence to the recommended 150 min of MVPA weekly (The Norwegian Directorate of Health, 2014; World Health Organization, 2010), without additional time-consuming exercise. Besides, decreased car use and increased everyday cycling could favor not only human health, but also environmental sustainability, through a reduction in greenhouse gas emissions (De Hartog et al., 2010; Woodcock et al., 2009), noise and pollution (de Nazelle et al., 2011). Therefore, enhancing the substitution of car commutes by more active modes is emphasized in transport policies, also in Norway (The Norwegian Ministry of Transport, 2017). Belgian GPS-data from >10,000 bike trips confirmed that e-bikes seem to represent a valid alternative for commuting, as the usage of e-bikes was higher on working days than on weekends, with longer distances cycled (Lopez et al., 2017). Also, in line with previous findings (Fyhri and Fearnley, 2015; Kroesen, 2017), e-bikes were used for longer distances than regular bikes, and trips up to 13 km seemed feasible for e-bike users (Lopez et al., 2017).

Regarding e-bike user characteristics, previous studies yield inconsistent findings (Fishman and Cherry, 2016). Qualitative, small-scale studies among e-bike owners (Dill and Rose, 2012; Popovich et al., 2014), have found those possessing e-bikes to be older, and with higher education and income than the average population, which also corresponds to recent Norwegian survey data (Simsekoglu and Klöckner, 2018). On the other hand, survey data on early adopters in Australia, reported e-bike owners to have lower education and income, yet higher age, than the population in general (Wolf and Seebauer, 2014). Other studies recruiting convenience samples from e-bike retailers (Hiselius and Svenssona, 2014; Sundfør and Fyhri, 2017), found the appeal to e-bikes to be stronger among those with little interest in, and low levels of PA in general, and also differing motivation for buying an e-bike between the younger and the elderly.

In sum, there is still a lack of large-scale analyses on the travel behavior of e-bikes (Fishman and Cherry, 2016), and current knowledge regarding who the e-bike users are, is inconsistent. Likely, commuters represent a user group with an unexploited potential when it comes to e-bike usage (Plazier et al., 2018). Based on this, together with the fact that no previous studies have addressed these issues among employees in a “real-life”- setting (i.e. under natural conditions), the aims of the present study were to assess:

1. Type of bike access (e-bike vs. regular) in relation to place of residence (i.e. county), sociodemographic variables (age, sex, educational level, income, ethnicity) and habitual physical activity level.
2. Whether e-bike owners cycle more often and longer distances to work than those possessing a regular bike only.

## 2 Methods and materials

### 2.1 Setting

The present study was conducted in three counties in Southern and Western Norway: Sogn og Fjordane, Vest-Agder and Aust-Agder (henceforth treated together as Agder). Førde, being the most densely populated municipality in Sogn og Fjordane, was in 2012 allocated $ 1 billion NOK (about 107 billion EUR) to develop the infrastructure over a period of 8 years from 2017, to promote active transport and a more sustainable traffic situation. The Agder counties, incorporating Kristiansand Municipality, are used as comparison. Kristiansand is the fifth largest city in Norway, and the city with the greatest bicycle rate, entailing a 10% share of all travels (Institute of Transport Economics, 2014). In addition, geography and climate differs between Western and Southern Norway, with the West being generally hillier, and the climate being wetter and windier.

### 2.2 Study design and sample

During the spring and autumn of 2017, a web-based questionnaire survey was conducted among public employees in Sogn og Fjordane and Agder. The aim was to invite all public employees in these counties, and possible respondents were identified through their institution. From a list of all public institutions, contact persons, councillors, public health coordinators and IT-employers were contacted by e-mail, with request to provide employees' e-mail addresses (74.500 eligible public employees). By unclear or no response, the institution was contacted by phone to clarify inclusion or not. By positive response, e-mail addresses and names of employees were collected. In total 76 institutions signed up, and 27.663 e-mail addresses were obtained. In addition, 13 institutions wanted to sign up, but rejected to provide employees' e-mail addresses. For these institutions, a separate link was assigned for survey access, using the institution's identity number. A total of 10.634 potential respondents were given access by their institution. In sum, 38.297 public employees obtained access to the questionnaire survey, either by personal e-mail including a unique link, or by open-link forwarded from their institution.

The survey was first distributed in Sogn og Fjordane in the spring of 2017, and then in Agder in the autumn of 2017. The Norwegian centre for research data (NSD) conducted the survey through their service of NSD web-survey. All potential respondents received invitation to participate and study information either by personal email or by email forwarded from their institution. Concerning incentives, respondents in Sogn og Fjordane were in the draw of an I-pad, while in Agder respondents located at one institution were in the draw of 10 vouchers valued 500 NOK each, for use in the institution's cafeteria. Each respondent provided informed consent by entering the questionnaire. After a first release of the survey, two reminders were sent by e-mail with two weeks intervals. Further, the questionnaire was closed two weeks after the last reminder. For the 13 institutions that did not provide e-mail addresses, a contact person was responsible for distributing the link to the questionnaire, either through a personal e-mail to the employees, or through the intranet. The same procedure was repeated three times, with two weeks intervals. A total of 3540 (9.2% of those given access to the survey) participants entered the survey questionnaire. To be included in the initial analyses, participants had to answer a question on bike access. After removing one participant for obvious incorrect completion, the study sample consisted of 1977 (5.2% of those given access to the survey, 55.8% of those entering the questionnaire) subjects. The study was approved by the Regional Committee for Medical and Health Research Ethics (REK) with reference number 2016/1897/REK vest.

### 2.3 Measurements

The questionnaire assessed cycling frequency, correlates of cycling, leisure time PA level, health status comprising quality of life (physical, mental, social, and economical dimensions), and background variables such as age, sex, income, educational level, and place of residence (municipality). Also, car access (including number of cars), bike access, bike type, frequency of commuting to work by bike, and distance from home to the workplace, was mapped.

The item “Do you have bike access?” categorized participants into “bike access” and “no bike access”. Further, type of bike was assessed asking “What kind of bike do you have?”, with the response alternatives (multiple answers were possible) “racing bike”, “mountain bike”, “hybrid bike”, “city bike”, “transport/cargo bike (non-powered)”, “transport/cargo bike (e-bike)”, “e-bike” and “other”. Those reporting “transport/cargo bike (e-bike)” and “e-bike” were categorized as “e-bike”, resulting in a trichotomized bike access variable; “regular bike” [1], “e-bike” [2] and “no bike-access” [3].

Number of days per week cycling to the workplace (possible range 0 to 5), was assessed for the four yearly seasons (autumn, winter, spring and summer) separately. Next, average frequency across season was calculated, hence possible scoring per week ranged from 0 to 5. The variable “cycling per week” was then trichotomized and coded into “do not cycle to work (0 days/week)” [0], “cycles occasionally (>0-2.5 days/week)” [1] and “cycles most of the time (>2.5-5 days/week)” [2].

One open-ended question assessed commuting distance; “How far is it from your home to the workplace?” which was trichotomized into “short distance (1-3 km)” [0], “medium distance (4-10 km)” [1] and “long distance (11-25 km)” [2]. Those reporting (i) not to cycle to work (*n* = 810), and (ii) residing shorter than 1 km and longer than 25 km from the workplace, i.e. outside a reasonably normal cycling distance (*n* = 393), were excluded.

Sociodemographic variables such as age, sex and county were mapped with straightforward questions. Ethnicity was determined from the question “Are both of your parents born in Norway?” and was further dichotomized into “no” [0] and “yes” [1].

Educational level was assessed asking “What is your highest completed education?”, with the six response alternatives; “less than 7 years of elementary school”, “primary school, 7-10 years”, “middle school”, “matriculation, economical upper secondary school, general upper secondary level”, “college/university, less than 4 years”, and “college/university, 4 years or more”. Further, educational level was dichotomized into “lower: less than college/university” [0] and “higher: college/university or more” [1]. Household income was assessed by an open-ended question, and further dichotomized into low [0] or high [1] household income, with cut-off determined by the median; 950.000 NOK (≈ 100.000 EUR).

Habitual PA level was assessed asking “Specify the level of PA in your leisure time?”, followed by four response alternatives; “reading, watching television or other sedentary activity”, “walking, cycling or moving for at least 4 hours”, “exercise, strenuous gardening or similar activities” or “vigorous exercise or participating in competitive sports regularly”(Saltin and Grimby, 1968). The variable was dichotomized into “reading and walking = low PA” [0] and “sport for exercise and strenuous exercise = high PA” [1], respectively.

### 2.4 Statistical analyses

The statistical analyses were performed using the statistical software package IBM SPSS Statistics version 24.0 (IBM Corp., Somers, New York, USA). Descriptive analyses were conducted, using One-Way Analysis of Variance (ANOVA) for continuous variables and Chi-square tests for categorical variables. Descriptive data are presented as means and standard deviations (SD) for continuous variables, and as numbers and proportions for categorical variables. A two-sided *p*-value of ≤0.05 was considered statistically significant.

Binary logistic regression analyses were conducted to explore associations between county (Agder vs. Sogn og Fjordane), sociodemographic variables (age, sex, educational level, income, ethnicity) and habitual PA level, with type of bike (e-bike vs. regular bike), among those reporting bike access (*n* = 1522). A multiple logistic regression model was fitted, including all variables simultaneously (Table 1). Further, stepwise multinomial logistic regression analyses were performed (Hosmer Jr et al., 2013) to calculate relations between type of bike (e-bike vs. regular bike) and (i) cycling frequency to work (occasionally and most of the time vs. never), among those reporting bike access (*n* = 1655), and (ii) cycling distance to work (medium and long distance vs. short distance), among those reporting to cycle to work, and to reside >1 km and ≤ 25 km from the workplace (*n* = 571). Firstly, binary relations between the variables county, age, sex, educational level, income, ethnicity and habitual PA level with the two outcomes, i.e. (i) cycling frequency and (ii) cycling distance was assessed, to determine which variables to include in the multivariate models (*p* = ≤0.3). Secondly, multivariate models were performed, excluding non-significant variables (*p* = ≤0.05) stepwise backwards. Hence, the final model for the outcome cycling frequency included the variables ethnicity, educational level, and habitual PA level, in addition to bike type, as main effects (Table 2). The final model for the outcome cycling distance included county and gender, in addition to bike type, as main effects (Table 3). Further, to assess potential interaction effects between bike type and the covariates, interaction terms were included in the models one by one, i.e. bike type*ethnicity, bike type*educational attainment, and bike type*habitual PA-level for cycling frequency, and bike type*county, and bike type*sex for cycling distance. Results from the logistic regression analyses are presented as odds ratios (OR) with 95% confidence intervals (CI).

**Table 1:** Bike ownership (e-bike vs. regular bike) in relation to county, sex, ethnicity, age, educational level, income and habitual physical activity level (*n* = 1522).

Total	E-bike vs. regular bike OR (95% CI)
County (Agder vs. Sogn og Fjordane)	3.98⁎ (2.53–6.26)
Sex (women vs. men)	1.03 (0.72–1.48)
Ethnicity (native vs. non-native)	0.73 (0.41–1.29)
Age (years)#	1.01 (0.99–1.03)
Educational level (high vs. low)	1.04 (0.63–1.71)
Income (high vs. low)	1.32 (0.92–1.89)
Physical activity level (high vs. low)	0.56⁎ (0.39–0.82)

Regular bike as reference group.

⁎ P = ≤0.05. ORs and 95% CIs were determined from binary logistic regression analyses.

# Continuous variable.

**Table 2:** Cycling frequency in relation to bike type, adjusted for ethnicity, educational level, and habitual physical activity level as main effects (*n* = 1655).

Total	Cycles occasionally OR (95% CI)±	Cycles most of the time OR (95% CI)±
Type of bike (e-bike vs. regular bike)	3.71⁎ (2.44–5.65)	4.28⁎ (2.79–6.55)
Ethnicity (non-native vs. native)	1.68⁎ (1.11–2.56)	1.96⁎ (1.27–3.00)
Educational level (low vs. high)	0.48⁎ (0.32–0.71)	0.77 (0.53–1.11)
Physical activity level (low vs. high)	0.73⁎ (0.57–0.93)	0.72⁎ (0.55–0.94)

± Cycles occasionally: >0–2.5 days per week, cycles most of the time: >2.5–5 days per week. Reference group; those reporting to never commute by bike (0 days per week).

⁎ P = ≤0.05. Multinomial logistic regression analyses were used to calculate ORs and 95% CIs.

**Table 3:** Cycling distance to work (among those cycling to work and residing >1 km and ≤ 25 km from the workplace) in relation to type of bike, adjusted for county and sex as main effects (*n* = 571).

Total	Medium distance± OR (95% CI)	Long distance± OR (95% CI)
Type of bike (e-bike vs. regular bike)	1.74⁎ (1.04–2.90)	1.76 (0.92–3.38)
County (Sogn og Fjordane vs. Agder)	0.73 (0.49–1.09)	0.46⁎ (0.25–0.83)
Sex (men vs. women)	1.11 (0.76–1.62)	2.21⁎ (1.36–3.63)

± Medium distance: 4–10.9 km, long distance: 11–25 km. Reference group; those cycling short distance (1–3.9 km) to work.

⁎ P = ≤0.05. Multinomial logistic regression analyses were used to calculate ORs and 95% CIs.

## 3 Results

In total 1977 participants were included in the initial analyses; 56.9% from Agder, 63.9% women, 90.8% native Norwegians, 83.0% with higher education, and 42.1% with higher household income. Further, 1768 (89.4%) subjects reported bike access, of whom 158 (8.0%) had access to an e-bike (Supplemental Table 1).

Results from the binary logistic regression analyses showed that respondents possessing an e-bike were less likely to report high levels of leisure time PA (OR 0.56 (CI 0.39-0.82)), compared to those possessing a regular bike only (Table 1). For those residing in Agder, the likelihood of possessing an e-bike (vs. regular bike) was almost 4 times higher (OR 3.98 (CI 2.53-6.26)), compared with participants residing in Sogn og Fjordane. No significant differences were found for sex, ethnicity, age, educational level or income across bike ownership.

For the multinomial logistic regression analyses, the OR for cycling occasionally and for cycling most of the time (vs. never commute by bike) was 3.71 (CI 2.44-5.65) and 4.28 (CI 2.79-6.55), respectively, for e-bike owners compared to those possessing a regular bike only, after adjusting for ethnicity, educational level and habitual physical activity level (Table 2). Also, a significant interaction was observed between type of bike and ethnicity for cycling most of the time vs. never commute by bike. The OR for cycling most of the time with an e-bike (vs. regular bike) was 0.75 for non-natives and 5.18 for natives. No interaction effect was found for type of bike and ethnicity for cycling occasionally vs. never commute by bike.

For cycling distance, the multinomial logistic regression analyses revealed that e-bike users were more likely to cycle medium distance to work (vs. short distance), compared with those using a regular bike (OR 1.74 (CI 1.04-2.90)), when adjusting for county and sex as main effects (Table 3). For cycling medium distance (vs. short distance) to work, an interaction effect was found for bike type and sex. The OR for cycling medium distance with an e-bike (vs. regular bike) was 3.99 for men and 1.23 for women. For cycling long distance (vs. short distance) to work, no significant main effects for type of bike was found, nor any significant interaction between type of bike and sex.

## 4 Discussion

To our best knowledge, no previous studies have provided population data on commuting frequency and commuting distance across e-bike and regular bike ownership among adults in a “real-life”- setting, i.e. under natural conditions. In the present study, assessing active transport to work among public employees residing in Western and Southern Norway, e-bike owners reported to cycle more often and longer distances to work than those possessing a regular bike only. We also found that e-bike owners reported lower levels of leisure time PA, and most of them were residing in Southern Norway, compared with Western Norway.

The more frequent use of an e-bike, and longer cycling distances, may be due to the potential to overcome typical barriers for cycling, when riding an e-bike compared with riding a regular bike. Several studies have shown that most of the early adopters of e-bikes were people with disabilities and increasing age (Dill and Rose, 2012; Hiselius and Svenssona, 2014; Popovich et al., 2014; Wolf and Seebauer, 2014). More recently though, e-bikes seem to appeal to a broader group, as barriers such as topography, distance, time use and the ability to ride a bike without needing to shower afterwards, are commonly mentioned to be reduced (Cairns et al., 2017; Fyhri and Fearnley, 2015; Kroesen, 2017). Concerning time use, e-bikes are found to be speed competitive with both public transport (Plazier et al., 2017) and private cars (B. Gojanovic et al., 2011), meaning that cycling with an e-bike does not necessarily entail longer travel time than using public transport or driving a car. In line with our results, a review from 2015 suggested that e-bike users cycle more frequently, and also travel longer distances, than those riding a regular bike (Fishman and Cherry, 2015). More specifically, Fyhri and Fearnley (2015) reported number of bike trips to increase from 0.9 to 1.4 per day, cycling distance to increase from 4.8 km to 10.3 km, and e-biking as share of all transport to increase from 28% to 48%, when e-bike access was provided for 2–4 weeks. In the Netherlands, Kroesen (2017) performed a conceptual model based on data from three national mobility surveys and found that average distance cycled with an e-bike was 3.0 km, compared to 2.6 km with a regular bike. Moreover, Lopez et al. (2017) found that e-bikes were used for longer distances than traditional bikes, when analyzing GPS data from >10,000 bike trips conducted in Flanders, Belgium. And, also among older e-bike users in Flanders, e-biking has shown to relate to higher probabilities and higher volumes of cycling, both for transportation and recreation (Van Cauwenberg et al., 2018a, Van Cauwenberg et al., 2018b).

In the present study those classified as “low” PA level, i.e. those reporting to be less physically active during leisure time, were found to be overrepresented among e-bike owners. Accordingly, the strongest appeal to e-bikes in Norway has been found among those with little interest in and low levels of PA in everyday life (Sundfør and Fyhri, 2017), and the interest for buying an e-bike seems greater among those who have not cycled recently (Fyhri and Sundfør, 2014). In the present sample, we also found that native Norwegians using an e-bike had higher odds of cycling most of the time, compared with non-natives using an e-bike, despite greater cycling frequency in general in non-natives, both occasionally and most of the time. Although current evidence is unclear and mixed, studies have found active transportation to be influenced by ethnicity (Thern et al., 2015) and income (Heinen et al., 2010). Considering that significantly more native Norwegians were classified as high income (results not shown) in our study, more frequent cycling in general in non-natives, yet not with an e-bike, may be just as much an expression of socio-economics. Nonetheless, the fact that those reporting lower levels of habitual PA were overrepresented among the e-bike owners in our sample, demonstrates the potential of e-bikes to reach novel cyclists and facilitate reduced driving and increased cycling.

E-bikes could maintain or increase PA levels, and thus promote health, among those who do not consider traditional pedaling a realistic mode choice (Jones et al., 2016). For illustration, an American study providing 21 sedentary commuters with an e-bike for 4 weeks, found that e-bike access assisted the commuters in meeting the official PA guidelines, which in turn decreased cardio metabolic risk factors (Peterman et al., 2016). In addition, in a sample of older Flemish adults, the strongest associations between e-bike usage and cycling levels were found for females, those with cycling limitations, and those with higher BMI (Van Cauwenberg et al., 2018a, Van Cauwenberg et al., 2018b). In our study, both males and females had higher odds of cycling medium distance to work (compared with short distance) when using an e-bike (compared with a regular bike), yet with the most pronounced relations found for males, which was somewhat unexpected. Previous findings revealed that the e-bike resulted in considerably more cycling in females than males when measured as number of trips, yet not for mileage share of all transport (Fyhri and Fearnley, 2015). Men are repeatedly reported to cycle more than women (Heinen et al., 2010), especially in countries with low cycling rates and the lack of a mass-cycling culture (Aldred et al., 2016), which corresponds with our finding that males were more likely than females to cycle long distances, compared with short distances. Concerning bike type, on the other hand, older e-bike owners are previously found more likely to be females (Van Cauwenberg et al., 2018a, Van Cauwenberg et al., 2018b), and e-bikes seem to appeal more to women (Fyhri and Sundfør, 2014; Kaplan et al., 2018). It has been suggested that women may put the instrumental value of bikes over the advantages related to PA, resulting in stronger preferences for the motorized assistance of e-bikes, reducing typical barriers for cycling (Aldred et al., 2016). In general, females have lower muscular strength than males, which may increase the perceived attractiveness of e-bikes (Van Cauwenberg et al., 2018a, Van Cauwenberg et al., 2018b). Still yet, these previous findings were not supported by our data. Taken together, however, it is reasonable to believe the e-bike could assist the e-bike users in the present study, reporting lower levels of habitual PA, to achieve the recommended PA level.

In total 11.8% of the participants in Southern Norway owned e-bikes, compared to only 3.0% of the participants in Western Norway. This disparity in e-bike ownership may partly be explained by various levels of developed infrastructure supporting cyclists, and maybe also different weather conditions. However, due to more hilly terrain and wet and windy climate in Western Norway, one could expect the need for an e-bike to be greater in the West. Possibly, lower population density in Sogn og Fjordane may influence mode choice. If living more rurally, entailing longer distances from the home to the workplace, a bike may not be perceived a realistic mode choice anyway, and especially not if car driving does not come with the drawback of traffic jam, which in turn would favor cycling instead of car use. Also, the assortment of e-bikes may be greater in the South, with more visible and more pushing retailers, yet these are only speculations.

### 4.1 Strengths and limitations

We included a rather large sample of public employees in three Norwegian counties, thus providing population data on e-bikes, which is currently scarce. To the authors´ best knowledge, no previous studies have assessed commuting by e-bike and regular bike among adults in a “real-life”- setting. As the target group was all public employees, recruitment was done through public institutions, not through bike retailers or any other sources targeting bike owners specifically, or those interested in becoming bike owners. We believe that such a broader recruitment strategy, i.e. inclusion by virtue of being a public employee, provided opportunities for obtaining population data on e-bikes to a greater extent than if applying a more confined recruitment strategy. Still yet, the low response rate reduced the potential of this broader recruitment strategy and resulted in reduced generalizability, as females, native Norwegians and those highly educated were overrepresented in the present study, compared with corresponding age groups in the general Norwegian population. Also, data collection was conducted in three out of Norway's nineteen counties, reducing generalizability further. Concerning the measure of habitual PA, this was rather inaccurate, hence challenging both the validity and reliability of the further classification into “high” and “low” PA level. In addition, different sizes of sub-groups, especially native Norwegians (91.1%) vs. non-natives (8.9%) and the distribution of regular bikes (91.1%) vs. e-bikes (8.9%), decreased statistical power, which in turn may have hindered significant results. Besides, small subgroups (e.g. non-natives possessing an e-bike) could impede the validity of study results. Another limitation is the nature of cross-sectional data, as reversed causality cannot be ruled out. That is, we do not know if greater cycling frequency in e-bike owners resulted from a recent purchase of an e-bike, or if these respondents also cycled frequently before the e-bike was acquired. Also, a related issue is that the survey did not capture information on mode changes, e.g. transitions from car to e-bike, or from traditional bike to e-bike. Lastly, as the survey was disseminated in Sogn og Fjordane in the spring of 2017, and in Agder in the autumn of 2017, the very rapid increase in sales numbers of e-bikes could have affected the significantly greater number of e-bike owners in Agder.

## 5 Conclusion

In the present sample of public employees residing in Southern and Western Norway, we found that e-bike owners cycled more often and longer distances to work than those possessing a regular bike only. Considering that e-bike owners reported lower levels of habitual PA than those possessing a regular bike only, it is possible that e-bike usage could result in increased PA levels. Also, notable differences in e-bike access were observed between counties, with more e-bike owners in Agder than in Sogn og Fjordane.

The following is the supplementary data related to this article.

Supplemental Table 1 Descriptives of the total sample (*n* = 1977) and sociodemographics and general physical activity level, according to bike ownership, distance to work and cycling frequency to work. Supplemental Table 1

## Declarations

Ethics approval and consent to participate

The study was approved by the Regional Committee for Medical and Health Research Ethics (REK) with reference 2016/1897/REK vest*.*

## Consent for publication

Not applicable*.*

## Availability of data and material

The data that support the findings of this study are available from the authors upon reasonable request.

## Competing interest

The authors declare that they have no competing interests.

## Funding

This research did not receive any specific grant from funding agencies in the public, commercial, or not-for-profit sectors.

## Authors' contributions

ABJ, EB and HBB designed the present study. ABJ and HBB analyzed the data and drafted the manuscript in collaboration with EB. All authors; SN, AS, LBA and AR have been involved in the development, coordination and/or implementation of the FACT-study. All authors have read and approved the final manuscript.
