# Supplementary material for: Public employees in South-Western Norway using an e-bike or a regular bike for commuting – A cross-sectional comparison on sociodemographic factors, commuting frequency and commuting distance
Source: Prev Med Rep. 2019 Apr 29;14:100881. doi: 10.1016/j.pmedr.2019.100881 (PMC6503162; doi:10.1016/j.pmedr.2019.100881)
Supplement: Supplemental Table 1 — Descriptives of the total sample (n = 1977) and sociodemographics and general physical activity level, according to bike ownership, distance to work and cycling frequency to work. [file mmc1.docx]

|  | Total | Regular bike  *n* (%) | E-bike  *n* (%) | Does not own a bike  *n* (%) | p-value* | Short^±^ distance  *n* (%) | Medium^±^ distance  *n* (%) | Long^±^ distance  *n* (%) | p-value* | Never bikes to work  *n* (%) | Bikes^#^ occasionally  *n* (%) | Bikes most^#^ of the time  *n* (%) | p-value* |
| --- | --- | --- | --- | --- | --- | --- | --- | --- | --- | --- | --- | --- | --- |
| Total |  | 1610 (81.4) | 158 (8.0) | 209 (10.6) |  | 287 (48.7) | 212 (36.0) | 90 (15.3) |  | 1271 (64.3) | 385 (19.5) | 321 (16.2) |  |
| County  Sogn og Fjordane  Agder | 853 (43.1)  1124 (56.9) | 717 (84.1)  893 (79.4) | 25 (3.9)  133 (11.8) | 111 (13.0)  98 (8.7) | **<0.001** | 105 (57.7)  182 (44.7) | 59 (32.4)  153 (37.6) | 18 (9.9)  72 (17.7) | **0.006** | 581 (68.1)  690 (61.4) | 147 (167.2)  238 (21.2) | 125 (14.7)  196 (17.4) | **0.008** |
| Gender  Women  Men | 1264 (63.9)  677 (34.2) | 1017 (80.5)  564 (83.3) | 100 (7.9)  56 (8.3) | 147 (11.6)  57 (8.4) | 0.09 | 183 (51.0)  97 (44.5) | 134 (37.3)  74 (33.9) | 42 (11.7)  47 (21.6) | **0.006** | 838 (66.3)  410 (60.6) | 238 (18.8)  140 (20.7) | 188 (14.9)  127 (18.8) | **0.03** |
| Ethnicity  Native  Non-native | 1796 (90.8)  175 (8.9) | 1469 (81.8)  135 (77.1) | 140 (7.8)  18 (10.3) | 187 (10.4)  22 (12.6) | 0.31 | 254 (48.4)  32 (50.8) | 194 (37.0)  18 (28.6) | 77 (14.7)  13 (20.6) | 0.29 | 1172 (65.3)  94 (53.7) | 342 (19.0)  42 (24.0) | 282 (15.7)  39 (22.3) | **0.008** |
| Age  (Mean (SD))** | 48.2 (11.1) | 48.4 (10.9) | 49.2 (10.9) | 46.6 (12.6) | 0.06 | 48.9 (10.7) | 48.5 (10.7) | 49.5 (10.8) | 0.77 | 47.9 (11.3) | 48.7 (10.8) | 49.1 (10.8) | 0.14 |
| Education  Less than college  College or more | 324 (16.4)  1640 (83.0) | 245 (75.6)  1358 (82.8) | 23 (7.1)  134 (8.2) | 56 (17.3)  148 (9.0) | **<0.001** | 36 (50.7)  250 (48.4) | 23 (32.4)  188 (36.4) | 12 (16.9)  78 (15.1) | 0.79 | 242 (74.7)  1018 (62.1) | 36 (11.1)  349 (21.3) | 46 (14.2)  273 (16.6) | **<0.001** |
| Income  < 950 000 NOK  > 950 000 NOK | 1049 (53.1)  832 (42.1) | 825 (78.6)  709 (85.2) | 79 (7.5)  78 (9.4) | 145 (13.8)  45 (5.4) | **<0.001** | 149 (50.0)  124 (45.9) | 104 (34.9)  102 (37.8) | 45 (15.1)  44 (16.3) | 0.62 | 699 (66.6)  506 (60.8) | 205 (19.5)  166 (20.0) | 145 (13.8)  160 (19.2) | **0.004** |
| Physical activity level  Read and stroll  Recreational sports and strenuous exercise | 1102 (55.7)  759 (38.4) | 867 (78.7)  646 (85.1) | 108 (9.8)  44 (5.8) | 127 (11.5)  69 (9.1) | **0.001** | 155 (49.4)  124 (48.6) | 119 (37.9)  83 (32.5) | 40 (12.7)  48 (18.8) | 0.10 | 727 (66.0)  450 (59.3) | 203 (18.4)  169 (22.3) | 172 (15.6)  140 (18.4) | **0.01** |
| Distance (km) from home to work among those cycling to work  (Mean (SD))** | 5.7 (5.3) | 5.5 (5.4) | 6.5 (5.2) |  | 0.11 |  |  |  |  |  |  |  |  |
| Frequency (d/week) of cycling to work  (Mean (SD))** | 1.0 (1.6) | 0.9 (1.5) | 1.9 (1.8) |  | **<0.001** |  |  |  |  |  |  |  |  |

**Supplemental table 1:** Descriptives of the total sample (*n*=1977) and sociodemographics and general physical activity level, according to bike ownership, distance to work and cycling frequency to work.

*****P-values were calculated using Chi square tests (categorical variables) and One-way ANOVA (continuous variables). Significance level p = ≤0.05 (presented in **bold)**. ****** Continuous variables are presented as mean and standard deviation (SD)

^±^Short distance: 1-3 km, medium distance: 4-10 km, long distance: 11-25 km. Those living <1 km and >25 km from the workplace, in addition to those reporting not to cycle, were excluded *(n*=589)

^#^ Cycles occasionally: >0-2.5 days per week, cycles most of the time: >2.5-5 days per week.
